# The first report on a new *Tor* species, *Tor barakae* Arunkumar & Basudha 2003, from Bangladesh using DNA barcoding technique

**DOI:** 10.1016/j.heliyon.2023.e21764

**Published:** 2023-10-28

**Authors:** Md Amdadul Haque, Jonaira Rashid, Md Lipon Mia, Md Khaled Rahman, Md Azhar Ali, Anuradha Bhadra, Yahia Mahmud

**Affiliations:** a Bangladesh Fisheries Research Institute, Mymensingh, 2201, Bangladesh; b Bangladesh Fisheries Research Institute, Freshwater Station, Mymensingh, 2201, Bangladesh; c Bangladesh Fisheries Research Institute, Riverine Sub-station, Rangamati, 4500, Bangladesh

## Abstract

Mahseer are large-scale fish of the Cyprinidae family that inhabit South and Southeast Asian mountainous streams, rivers, and reservoirs. *Tor tor* and *Tor putitora*, two species of the *Tor* genus, were reportedly found in Bangladesh. This study aimed to confirm the species level of specimens collected from the Sangu River. The collected samples were identified using the DNA barcoding technique, followed by amplifying 645 bp of the cytochrome oxidase c subunit 1 gene (COI) using the FishF1/FishR1 universal primer. The sequence similarity was conducted using BOLD and NCBI databases which showed 99.85–100 % similarity to the reference genome. The genetic divergence between *T. putitora* vs. SRI, BT, and ST was found to be 0.0239, 0.0239, and 0.0238, respectively. The genetic divergence between *T. tor* vs. SRI, BT, and ST was 0.0272, 0.0272, and 0.0270, respectively. In the phylogenetic tree, two clusters were formed where collected specimens (SRI, BT, and ST) formed a subcluster with the reference genome (NC_056296.1 *T. barakae*) with 100 % bootstrap support. This study's findings revealed the presence of a new *Tor* species named *Tor barakae* in the Sangu River basin in Bangladesh.

## 1 Introduction

Freshwaters in South and Southeast Asia are a haven for Mahseer fishes of the Cyprinidae family. Large-scale Mahseers are regarded as economically valuable for aquaculture, capture fisheries, and as sport fish. They are mainly found in mountain streams with rocky bottoms but can also be found in rivers and lakes [1,2]. There are a total of 59 mahseer species, among which 36 belong to the *Tor* genus, 22 to *Neolissochilus*, and one to *Naziritor* [3]. Only those species that belong to the genus *Tor* are referred to as the ‘true mahseers’ [4,5]. Similarly, 14 additional species of *Tor* were recognized in the *trans*-Himalayan region and Southeast Asia [4,6,2]. The maximum species diversity for the *Tor* genus in southern Asia was found in the Indian subcontinent, with seven species spread across it [1]. The two morphologically studied species of the *Tor* genus, named *Tor tor* and *Tor putitora*, are reportedly found in the mountainous streams of Sylhet, Netrokona, Mymensingh, Bandarban, and the Kaptai reservoir in Bangladesh [7]. Mahseer's identity remained taxonomically ambiguous, particularly when considering its morphological characteristics [4]. Taxonomists dispute the recognized morphological criteria for classifying the mahseer species, such as the relationship between head length and body depth [8] or the anatomy of the lip and median lobes [9]. The integration of morphological and molecular techniques is crucial for accurately determining the taxonomic classification and conservation status of *Tor* species. DNA barcoding is a modern molecular technique capable of successfully identifying fish species while revealing intra- and interspecific variation [10]. If properly applied, it can eliminate current fish misidentification and the availability of cryptic species that imitate and, at the same time, impede research, fishery management, and conservation efforts [11,12].

During the survey of hill stream fish, mahseer fish were found in the Sangu River, with distribution in Andarmanik, Boro Modok, and Ligri areas of the Sangu River in the Thanchi upazila of Bandarban. The water in these regions is deep with sandy or gravelly bottoms, which provide a favorable environment for Mahseer inhabitation. Locally, Mahseer is also known as Mikimau or Phorong, which is morphologically different from the other two (*T. tor* and *T. putitora*) existing species. Lack of knowledge about biology, population, and distribution keeps it out of the realm of conservation management. This study aimed to identify mahseer fishes at the species level using morphological and molecular approaches.

## 2 Materials and methods

### 2.1 Sample collection and preservation

**Fig. 1:**
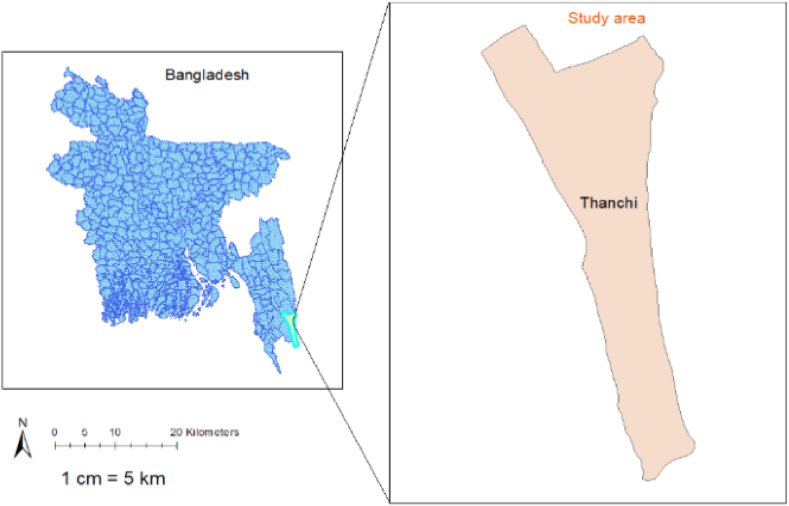
Sampling site (Sangu River, Thanchi Upazila, Bandarban).

**Fig. 2:**
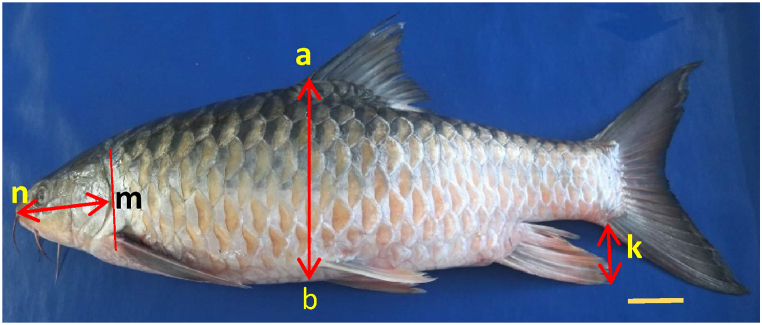
Morphometric variables differentiate the specimen from other *Tor* species. Where **nm** - head length, **ab**-body depth, and **k** - the caudal fin's tip. Scale: 5 cm.

Three morphologically distinct fish samples were collected from the Sangu River in the Thanchi upazila of Bandarban (Fig. 1). The samples were labeled as Sangu River 1 (SR1), Bandarban Tor (BT), and Sangu *Tor* (ST). A clear photograph was taken and presented in Fig. 2. For the molecular part of the study, each sample was thoroughly cleansed with distilled water to eliminate any potential impurities, followed by excising 100 mg of caudal fins using sterile scissors, appropriately labeled, stored in 95 % ethanol, and kept at −20 °C till further use. The ethical standards outlined for animal handling were strictly followed throughout the study while handling fish specimens.

### 2.2 Morphological approach

Morphometric and meristic characters of each fish were recorded by following the method reported by Gharaei [13] with slight modifications. The abbreviations used for different morphological characteristics were total length (TL), standard length (SL), head length (HL), body depth (BD), dorsal fin height (DFH), pectoral fin height (PFH), pelvic fin height (PeFH), and anal fin height (AFH), respectively.

### 2.3 Molecular approach

#### 2.3.1 DNA extraction and PCR amplification

DNA extraction from the caudal fin tissue was performed using the PureLink (TM) Genomic DNA Mini Kit (Invitrogen by Thermo Fisher Scientific, USA). For DNA barcoding, partial 5′ regions of the COI gene were amplified in a final volume of 50 μL with added 25 μL DreamTaq (TM) PCR Master Mix (Thermo Fisher Scientific, USA), 1 μL of each primer (10 μM), 22 μL PCR-grade distilled water, and 100 ng DNA. The primers used for the amplification of the COI gene were-

F1: 5′-TCAACCAACCACAAAGACATTGGCAC-3′

R1: 5′-TAGACTTCTGGGTGGCCAAAGAATCA-3′ [14].

The thermal cycle sequences used to conduct each reaction in a 96-well thermal cycler (ASTEC Thermal Cycler GeneAtlas, Japan) were: 2 min for initial denaturation at 95 °C, 35 cycles comprised at 94 °C for 35 s, annealing primer at 54 °C for 30 s, primer extension at 72 °C for 1 min, and final extension for 10 min at 72 °C. The COI-amplified products were separated using a 1.2 % agarose gel. A commercial gel purification kit (PureLinkTM PCR purification, Thermo Fisher Scientific, USA) was used to purify the PCR products, which were later sequenced by Apical Scientific Sdn Bhd, Malaysia.

### 2.4 Sequenced data analysis

After receiving the sequencing data, the raw data were manually checked and edited using the BioEdit program [15]. The basic local alignment search tool (BLAST), an online tool from the National Centre for Biotechnology Information (NCBI) to assess the homology with the GenBank data, was used to analyze the mtDNA COI gene nucleotide sequences. The nucleotide sequences were also compared to the barcode of life data (BOLD) database for species determination.

The ClustalW program included in MEGA-X was used to align the nucleotide sequences with each other [16], with a gap opening penalty of 15 and a gap extension penalty of 6.6. Using the maximum composite likelihood approach [17] and the Kimura-2 Parameter (K2P) model [18], the pairwise genetic distance between sample sequences was estimated in MEGA-X [18]. A phylogenetic tree was constructed using the maximum likelihood method [16,17] and the K2P model [18] to estimate the taxonomic relationship, with a bootstrap value of 1000 [19]. The phylogenetic tree was modified in iTOL v4 [20], followed by QR code generation using a QR code generator (https://goqr.me/).

## 3 Results

### 3.1 Morphological identification of the collected sample

The general morphological characteristic in terms of the body surface color of the collected specimens was darkish blue on the back with a reddish ventral side, with the anal fin of the fish reaching the caudal fin base (Fig. 2). Morphometric and meristic characters were enlisted in Table 1, Table 2. HL and BD ratios of the collected samples (SRI, ST, and BT) were 0.73:1, 0.66:1, and 0.69:1, respectively.

**Table 1:** Morphometric characters of the collected specimens.

Measurements (cm)	Range	Mean
TL	56.3–65.8	59.6 ± 4.39
SL	45.2–52.3	47.8 ± 3.91
HL	10.1–13.1	11.5 ± 1.51
BD	15.2–17.9	16.5 ± 1.36
DFH	10.4–11.8	10.9 ± 0.75
PFH	10.1–11.7	11.2 ± 1.65
PeFH	9.2–10.9	9.8 ± 0.95
AFH	9.0–10.8	9.7 ± 0.99

**Table 2:** Meristic characters of the collected specimens.

Characters	Number
Dorsal fin rays	12 (3/9)
Pectoral fin rays	15
Pelvic fin rays	10 (1/9)
Anal fin rays	9 (2/7)
Caudal fin rays	19
Lateral line scales	26–27

### 3.2 Molecular identification of the collected samples

A FishF1/FishR1 universal primer was used to amplify the mtDNA COI gene in three collected samples. After the PCR products were electrophoresed, the gel documentation system showed clear and distinct DNA bands, cementing the righteous PCR condition setting. The nucleotide sequence length of the three specimens was 645 bp, devoid of insertions, deletions, or stop codons in any of the sequences. The sequence similarity data using BOLD and NCBI databases are given in Table 3. Following submitting the nucleotide sequence to NCBI, the provided accession numbers for SR1, BT, and ST were OQ694386, OQ694387, and OQ694388.

**Table 3:** Nucleotide BLAST analysis with the reference genome to identify the samples.

Sample ID	Genbank (NCBI)		Genbank (BOLD)			Genbank (NCBI) accession no.
	Similarity (%)	Reference genome	Similarity (%)	Reference genome	–	–
*SRI*	99.85	NC_056296.1	100	MG518435	*Tor barakae*	OQ694386
BT	100	NC_056296.1	100	MG518435	*Tor barakae*	OQ694387
ST	100	NC_056296.1	100	MG518435	*Tor barakae*	OQ694388

### 3.3 Genetic diversity and divergence analysis

**Fig. 3:**
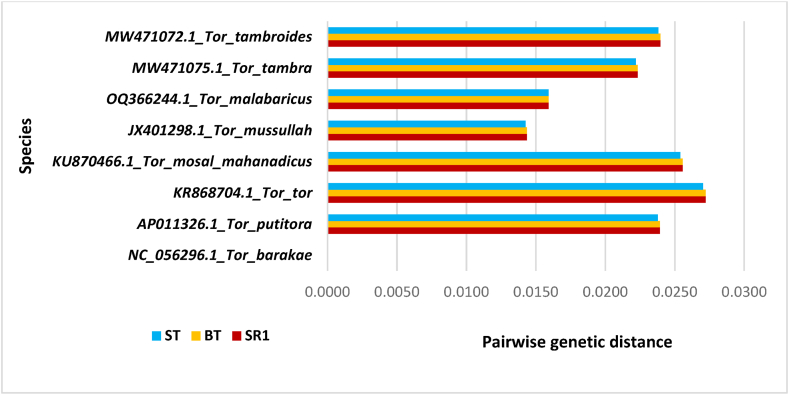
Pairwise genetic distance of collected specimens (*SRI*, BT, and ST) with the reference genome.

Pair-wise genetic distance was analyzed using the Kimura 2-parameter (K2P) model in the Mega X program, followed by comparing genetic distance with the reference genome of a voucher specimen collected from NCBI. The results indicated no genetic diversity among the collected samples (SRI, BT, and ST). However, a momentous divergence was observed when the samples were compared to the other seven species within the *Tor* genus. The slightest genetic divergence was found to be 0.0144, 0.0144, and 0.0143 for *T. mussullah* vs. SRI*,* BT, and ST. Similarly, the highest genetic divergence observed were 0.0272, 0.0272, and 0.0270 for *T. tor* vs. SRI*,* BT*,* and ST, respectively (Fig. 3).

### 3.4 Phylogenetic tree

**Fig. 4:**
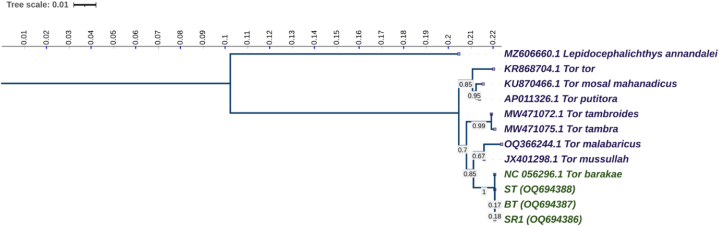
The maximum likelihood phylogenetic tree was reconstructed from the collected specimens (SRI*,* BT, and ST) with the reference genome.

The nucleotide sequences of collected samples were used to reconstruct a maximum likelihood phylogenetic tree. The Kimura 2-parameter (K2P) model with a 1000 bootstrap value was used to measure evolutionary genetic distance. In the phylogenetic tree, two clusters were formed where SRI, BT, and ST formed a sub-cluster with the reference genome (NC_056296.1 *T. barakae*) (Fig. 4) with 100 % bootstrap support and *Lepidocephalichthys annandalei* used as an outgroup.

### 3.5 QR code for the identified species

**Fig. 5:**
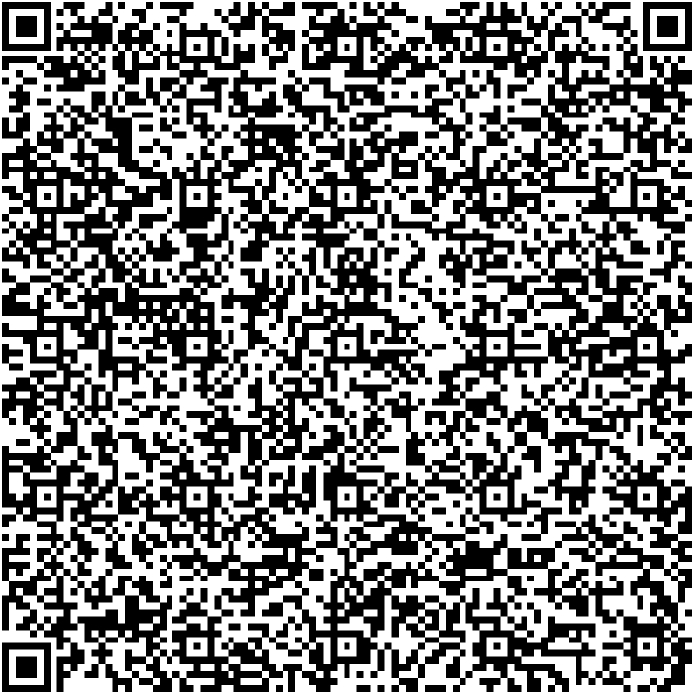
QR code of *T. barakae*.

A QR code was generated using a QR code generator (Fig. 5) to represent the identified fish species. The QR code scanning facilitated the accessibility of nucleotide sequences and comprehensive data of identified fish species.

## 4 Discussion

Mahseer species have negligible morphological differences, making distinguishing them challenging, thereby making conservation strategies difficult [2]. Various literary accounts are inconsistent or fail to account for the variety of species found in Indian mahseers [21]. Compared to BD, HL is significantly shorter in the case of *T. barakae* [[2], [22]]. The HL and BD ratios of SRI, BT, and ST were found to be 0.73:1, 0.66:1, and 0.69:1, respectively, suggesting significantly shorter HL than BD compared to *T. putitora*, with a ratio of 0.85:1 [23].

DNA-based identification approaches could be a crucial analytical substitute or complement to clarify the confusion, while DNA barcoding is a novel technique for swiftly, precisely, and automatically identifying various species. The COI barcoding region had been selected as the primary molecular diagnostic method for identifying animal species [24]. Data from mtCOI and mtCytb analysis using various species delimitation techniques also demonstrated the legitimacy of *T. barakae* species, which were evidently distinct from other congeners [2]. The average homology of mtCOI sequences was 99.95 % in BLAST, and 100 % in BOLD analysis (Table 3), which was reasonably satisfactory.

Low inter-species divergence (K2P) in the genus *Tor* has been reported earlier [25]. It was discovered that the genetic divergence between the species of the *Tor* genus ranged from 0.000 to 0.037 [6], respectively. Similarly, the genetic divergence between *T. barakae* and *T. tor* was reported to be 0.0263 [2]. In this study, the genetic divergence between *T. putitora* vs. SRI, BT, and ST was found to be 0.0239, 0.0239, and 0.0238. The genetic divergence between *T. tor* vs. SRI, BT, and ST was found to be 0.0272, 0.0272, and 0.0270, respectively, which corroborated well with previously published reports [6,2,25]. No genetic diversity among SRI, BT, and ST indicated that they are genetically uniform.

Two clusters were formed in the maximum likelihood phylogenetic tree reconstructed from the COI sample sequences (Fig. 4). SRI, BT, and ST formed clusters with the reference genome (NC_056296.1 *T. barakae*). Since no specimens formed a cluster with an outgroup, it indicated that SRI, BT, and ST were the *T. barakae* specimens. Individual samples are classified into phylogenetic branches per their taxonomic affinity, rendering members of the same species to be closely clustered [26].

The generated QR code provided the nucleotide sequence and comprehensive information about the detected fish, which can be easily read using a smartphone with a QR scanner without needing additional hardware. Regarding population, biology, and distribution at the micro level, *T. barakae* has yet to be well known. The species is listed as near threatened (NT) on the IUCN Red List due to its limited distribution and threats to its habitat in India [27]. Its population is also declining in Bangladesh due to overfishing, habitat destruction, and various other anthropogenic factors.

## 5 Conclusion

This study revealed that a novel *Tor* species, *T. barakae,* was found within the geographical confines of the Sangu River basin in Bangladesh. Further research is necessary to implement complete conservation strategies, such as habitat management, protection, and artificial reproduction.

## Author contribution statement

Md. Amdadul Haque, MS: Conceived and designed the experiments; Performed the experiments; Analyzed and interpreted the data; Wrote the paper. Jonaira Rashid, PhD: Conceived and designed the experiments; Performed the experiments; Analyzed and interpreted the data. Md. Lipon Mia, MS; Md.Azhar Ali, PhD: Performed the experiments. Md. Khaled Rahman, MS: Performed the experiments; Analyzed and interpreted the data. Anuradha Bhadra, PhD: Contributed reagents, materials, analysis tools or data. Yahia Mahmud, PhD: Conceived and designed the experiments; Contributed reagents, materials, analysis tools or data.

## Data availability statement

*Data associated with this study has been deposited at NCBI under* the accession number OQ694386, OQ694387, *OQ694388. Ethical statement*:

The animal study protocol was approved by the Institutional Review Board (Ethics Committee) of the Bangladesh Fisheries Research Institute (ac1590/BRFI).

## Funding statement

The author was grateful to the Bangladesh Fisheries Research Institute for providing funds for conducting the research.

## Declaration of competing interest

The authors declare the following financial interests/personal relationships which may be considered as potential competing interests:Md. Amdadul Haque reports was provided by Bangladesh Fisheries Research Institute. Md. Amdadul Haque reports a relationship with Bangladesh Fisheries Research Institute that includes: employment. Md. Amdadul Haque has patent pending to Md. Amdadul Haque. There are no conflicts of interest.
